# Identification of Region-Specific Cytoskeletal and Molecular Alterations in Astrocytes of *Mecp2* Deficient Animals

**DOI:** 10.3389/fnins.2022.823060

**Published:** 2022-02-15

**Authors:** Elena Albizzati, Elena Florio, Federica Miramondi, Irene Sormonta, Nicoletta Landsberger, Angelisa Frasca

**Affiliations:** ^1^Department of Medical Biotechnology and Translational Medicine, University of Milan, Milan, Italy; ^2^Division of Neuroscience, IRCCS San Raffaele Scientific Institute, Milan, Italy

**Keywords:** *MeCP2*, Rett syndrome (RTT), astrocyte, cytoskeleton, heterogeneity

## Abstract

Rett syndrome (RTT) is a neurodevelopmental disorder that represents the most common genetic cause of severe intellectual disability in females. Most patients carry mutations in the X-linked *MECP2* gene, coding for the methyl-CpG-binding protein 2 (MeCP2), originally isolated as an epigenetic transcriptional factor able to bind methylated DNA and repress transcription. Recent data implicated a role for glia in RTT, showing that astrocytes express *Mecp2* and that its deficiency affects their ability to support neuronal maturation by non-cell autonomous mechanisms. To date, some molecular, structural and functional alterations have been attributed to *Mecp2* null astrocytes, but how they evolve over time and whether they follow a spatial heterogeneity are two aspects which deserve further investigations. In this study, we assessed cytoskeletal features of astrocytes in *Mecp2* deficient brains by analyzing their arbor complexity and processes in reconstructed GFAP^+^ cells at different ages, corresponding to peculiar stages of the disorder, and in different cerebral regions (motor and somatosensory cortices and CA1 layer of hippocampus). Our findings demonstrate the presence of defects in *Mecp2* null astrocytes that worsen along disease progression and strictly depend on the brain area, highlighting motor and somatosensory cortices as the most affected regions. Of relevance, astrocyte cytoskeleton is impaired also in the somatosensory cortex of symptomatic heterozygous animals, with *Mecp2*^+^ astrocytes showing slightly more pronounced defects with respect to the *Mecp2* null cells, emphasizing the importance of non-cell autonomous effects. We reported a temporal correlation between the progressive thinning of layer I and the atrophy of astrocytes, suggesting that their cytoskeletal dysfunctions might contribute to cortical defects. Considering the reciprocal link between morphology and function in astrocytes, we analyzed the effect of *Mecp2* deficiency on the expression of selected astrocyte-enriched genes, which describe typical astrocytic features. qRT-PCR data corroborated our results, reporting an overall decrement of gene expression, which is area and age-dependent. In conclusion, our data show that *Mecp2* deficiency causes structural and molecular alterations in astrocytes, which progress along with the severity of symptoms and diversely occur in the different cerebral regions, highlighting the importance of considering heterogeneity when studying astrocytes in RTT.

## Introduction

Methyl-CpG-binding protein 2 (MeCP2), encoded by the X-linked *MECP2* gene, is an epigenetic regulator, named for its ability to bind methylated cytosines (Nan et al., 1997). It was initially described as a transcriptional repressor, diversely modulating gene expression depending on the cellular subtype and cerebral region, but it is now recognized that MeCP2 exerts multiple genome-wide regulatory activities (Jones et al., 1998; Nan et al., 1998; Tillotson and Bird, 2019). It can function as an organizer of chromatin architecture, a transcriptional activator, a regulator of mRNA splicing and miRNA processing, and we recently reported its role for proper primary cilium formation and functioning (Young et al., 2005; Chahrour et al., 2008; Skene et al., 2010; Szulwach et al., 2010; Bedogni et al., 2014; Frasca et al., 2020). MeCP2 is ubiquitously expressed throughout the body, but appears to be most abundant in brain, where its expression begins prenatally and progressively increases peaking during synaptic development and plasticity (Shahbazian et al., 2002). MeCP2 plays a critical role at different developmental stages and its importance for neuronal maturation and maintenance, circuit network and plasticity has been extensively described (Nguyen et al., 2012; Bedogni et al., 2014; Ip et al., 2018). Further, conditional animal models have been engineered to selectively silence *Mecp2* in different brain areas or neuronal subtypes. Studies using these mice reported that each mouse model develops a subset of RTT-like symptoms and provided useful information about the Mecp2 roles in different brain districts (Chen et al., 2001; Lindeberg et al., 2004; Fyffe et al., 2008; Samaco et al., 2009; Chao et al., 2010; Herrera et al., 2016). Although the full spectrum of MeCP2 functions remains to be elucidated, its importance for proper CNS functioning is highlighted by the existence of neurological disorders associated with *MECP2* mutations, among which Rett syndrome (RTT; OMIM 312750) is the most studied (Amir et al., 1999). RTT affects approximately 1 in 10,000 females worldwide, representing the most frequent cause of severe intellectual disability in girls and no cure is currently available. After an apparently normal development until 6–18 months of age, girls fail to reach developmental milestones and experience a rapid regression resulting in loss of acquired skills, profound intellectual disability, stereotypical movements, autistic features. Sensory and motor deficits are considered among the most debilitating symptoms in RTT patients, together with breathing alterations and seizures (Neul et al., 2010; Percy et al., 2010). *Mecp2* deficient mice recapitulate several clinical signs of RTT, exhibiting progressive defects in cognition and motor abilities, anxiety, breathing alterations and premature death (Guy et al., 2001; Cobolli Gigli et al., 2016). Although male hemizygous animals are widely used in basic research and pre-clinical studies due to their penetrant phenotype, it is strongly encouraged the investigations also in female heterozygous mice, which more closely resemble the human condition (Katz et al., 2012).

Among the neuropathological alterations reported both in RTT patient biopsies and in animal models, morphological studies revealed a reduction in brain volume and weight, mostly pronounced in the prefrontal, frontal and anterior temporal regions, with a preservation of cerebellum and occipital regions (Armstrong, 2005). In accordance with the higher expression of *Mecp2* in neuronal cells, the majority of defects accounts for morphological and functional impairments in neurons. Indeed, null neurons exhibit a reduction in soma size, dendritic arborization, spine density, and these changes appear area-specific, since they are mainly present in the frontal and temporal regions, including the motor, somatosensory cortex and hippocampal CA1 neurons (Kishi and Macklis, 2004; Armstrong, 2005; Fukuda et al., 2005; Belichenko et al., 2009; Chapleau et al., 2009; Tropea et al., 2009; Jentarra et al., 2010). Besides morphological alterations, *Mecp2* deficiency affects brain activity, disrupting the balance of synaptic excitation and inhibition with a pattern that varies among different brain regions (Dani et al., 2005; Calfa et al., 2015). However, *Mecp2* expression has been detected also in glial cells and, although in astrocytes its levels are five-fold lower than in neurons (Rusconi et al., 2008; Ballas et al., 2009; Maezawa et al., 2009; Olson et al., 2014), *in vitro* and *in vivo* studies reported that *Mecp2* deficiency in astrocytes contributes to the neuropathological manifestations of RTT. In fact, *Mecp2* null cortical astrocytes cause abnormal dendritic arborization in wild-type (WT) neurons and, similarly, mutant astrocytes differentiated from isogenic induced Pluripotent Stem Cells (iPSCs) from RTT patients adversely affect neuronal morphology (Ballas et al., 2009; Williams et al., 2014). Of relevance, evidence from conditional animal models highlights a role not only for neurons (Chen et al., 2001) but also for astrocytes (Lioy et al., 2011; Garg et al., 2015) in RTT pathophysiology. Indeed, the conditional reactivation of *Mecp2* only in astrocytes in an otherwise null animal ameliorates dendritic complexity, neuronal soma size and levels of vesicular glutamate transporter 1 (VGLUT1) and, at behavioral level, it leads to an improvement of locomotor and respiratory defects, prolonging lifespan (Lioy et al., 2011). Complementary, TAM-induced *Mecp2* conditional depletion in astrocytes mainly causes breathing alterations (Lioy et al., 2011; Garg et al., 2015).

Astrocytes are highly complex glial cells, which serve a multitude of roles spanning from supplying trophic and metabolic support to neurons, regulating neurotransmitter release, contributing to neuronal network activity and maintaining the integrity of the blood–brain barrier (BBB) (Barres, 2008; Sofroniew and Vinters, 2010; Verkhratsky and Nedergaard, 2018). Through the production of several bioactive molecules, astrocytes control different steps in brain development, including neuronal migration, synapse formation and elimination. Recent evidence highlighted astrocytes as heterogeneous cells within and among cerebral regions in terms of molecular, functional and morphological features (Bayraktar et al., 2015; Haim and Rowitch, 2017; Lanjakornsiripan et al., 2018; Ohlig et al., 2021). However, to the best of our knowledge, no RTT study did consider astrocyte heterogeneity. Further, only few reports analyzed which molecular mechanisms are compromised in *Mecp2* deficient astrocytes. In particular, a microarray study performed on cultured *Mecp2* null primary cortical astrocytes revealed 118 differentially regulated genes that might contribute to abnormal astrocyte signaling and neuronal support functions (Yasui et al., 2013). Similarly, microarray expression data of *Mecp2*^308/y^ cultured cortical astrocytes reported the alteration of 257 genes, generally involved in major cellular functions, including cell-cell communication and cellular development (Delépine et al., 2015). Curiously, transcriptional studies from Yasui’s and Delépine’s laboratories displayed very limited overlap, probably due to the use of different mouse models, different number of astrocyte passages *in vitro*, and/or to the presence of fetal bovine serum (FBS) in astrocyte cultures, with inter-vendor and inter-batch inconsistencies contributing to the variability. Further, a very recent proteomic analysis on the secretome derived from *Mecp2* knock-out (KO) cortical astrocytes revealed several deregulated proteins, suggesting the importance of non-cell autonomous mechanisms for neuronal maturation (Ehinger et al., 2021). Comparing data from the above-mentioned -omic studies, lipocalin 2 emerged as an interesting candidate and its addition to *Mecp2* null neurons rescued morphological defects (Yasui et al., 2013; Delépine et al., 2015; Ehinger et al., 2021). *In vivo*, a multi-omic study combining RNA sequencing and proteomic analyses of the adult *Mecp2* null cortex (P60) reported the alteration of 46 astrocyte-specific genes associated with astrocyte maturation and morphology, together with decreased levels of astrocytic proteins involved in apoptosis (Pacheco et al., 2017). Although these data highlight the dysregulation in RTT of relevant astrocytic genes, it is not known when these defects appear, if they homogenously occur in brain regardless of the cerebral region and if they are accompanied by morphological defects.

It has been suggested that astrocyte morphology might redirect their role and function, and changes in astrocyte structure, such a withdrawal or outgrowth of astrocyte processes, can modify their ability to interact with neurons (Verkhratsky et al., 2014; Schiweck et al., 2018). Loss of astrocyte complexity is a common pathological feature in many neurological disorders (Burda and Sofroniew, 2014) and, in the field of RTT research, fewer and poorly branched astrocytic ramifications have been described in the dentate gyrus and corpus callosum of *Mecp2^308/y^* mouse models as well as in the hippocampus of a conditional mouse, in which *Mecp2* has been postnatally inactivated (De Filippis et al., 2012; Nguyen et al., 2012). Further, cultured astrocytes derived from both *Mecp2* KO and *Mecp2*^308/y^ mouse brain and human RTT iPSCs displayed altered microtubule dynamics, which might influence the correct remodeling of the cytoskeleton and cellular shape (Nectoux et al., 2012; Delépine et al., 2016).

In this study, we analyzed the complexity of GFAP-stained astrocytes in three cerebral regions known to be affected in RTT (Fukuda et al., 2005; Belichenko et al., 2009; Chapleau et al., 2009; Tropea et al., 2009; Jentarra et al., 2010), i.e., the dorsal hippocampus and the somatosensory and motor cortices of WT, *Mecp2* null and heterozygous animals, using an optimized *Simple Neurite Tracer* Plugin by ImageJ (Tavares et al., 2017). Our results showed that *Mecp2* deficiency impairs astrocyte complexity along disease progression and that the effects vary depending on the specific cerebral region, particularly impinging on the motor and somatosensory cortex. Molecular studies reported an overall deregulation of astrocyte-enriched genes involved in several biological functions, which largely parallel the onset of cytoskeletal defects. Of relevance, analysis on the *Mecp2* heterozygous cortex highlighted the occurrence of non-cell autonomous effects as astrocytes expressing the WT allele exhibited comparable or more marked alterations than those expressing the mutant one.

## Materials and Methods

### Animals

The *Mecp2^tm1^*.^1*Bird*^ mouse strain was originally purchased from the Jackson Laboratories, then backcrossed and maintained on a clean CD1 background (Cobolli Gigli et al., 2016). These mice recapitulate the typical phenotype of C57BL/6 mice, with the advantage of having a larger progeny and minor risk of litter cannibalization. *Mecp2* null mouse genotype was determined by PCR on genomic DNA purified from ears using the following primers: 5′-ACCTAGCCTGCCTGTACTTT-3′, forward primer for the null allele; 5′-GACTGAAGTTACAGATGGTTGTG-3′, forward primer for the wild type allele; 5′-CCACCCTCCAGTTTGGTTTA-3′, common reverse primer.

*Mecp2* null mice at post-natal days (P) 20, 40, and 70, heterozygous female mice at P90 and P180, and the corresponding gender-matched WT littermates were used for immunofluorescence and molecular analyses (Table 1). Animals of each experimental group derived from at least three different litters and were sacrificed by rapid decapitation or by transcardial perfusion depending on experimental needs. Mice were housed in a temperature- and humidity-controlled environment in a 12 h light/12 h dark cycle with food and water *ad libitum*. All procedures were performed in accordance with the European Union Communities Council Directive (2010/63/EU) and Italian laws (D.L.26/2014). Protocols were approved by the Italian Minister for Scientific Research and by the San Raffaele Scientific Institutional Animal Care and Use Committee in accordance with the Italian law.

**TABLE 1:** Animals used in the study.

GFAP immunostaining and cortical thickness			qRT-PCR		
	P20	P40	P70	P20	P40	P70
♂ WT	5	4	5	8	8	8
♂ *Mecp2* KO	6	6	6	8	8	8

	**P90-110**		**P180**			

♀ WT	4		4			
♀ *Mecp2* HET	4		4			

### Immunofluorescence on Brain Sections

**FIGURE 1:**
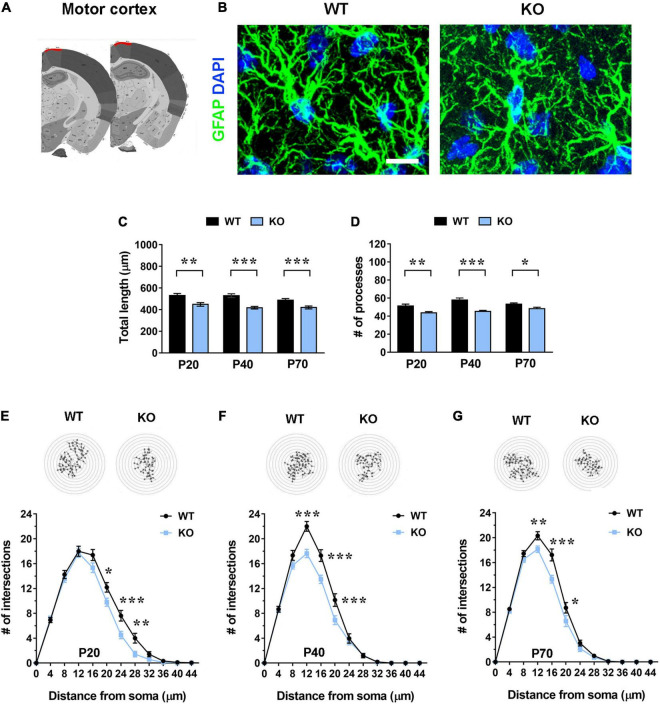
*Mecp2* KO astrocytes in the motor cortex (layer I) exhibit cytoskeletal atrophy in early-symptomatic (P20) as well as in moderate (P40) and severe symptomatic animals (P70). **(A)** Representative images of brain coronal sections, with the motor cortex (layer I) highlighted in red. **(B)** Micrographs are representative images of WT and *Mecp2* KO astrocytes immunostained for GFAP (green) and DAPI (blue) in the motor cortex (layer I) at P20. Scale bar = 10 μm. **(C,D)** The graphs show the total length of processes **(C)** and their number **(D)** in astrocytes in the motor cortex of WT and *Mecp2* KO mice at P20, P40, and P70. Data are represented as mean ± SEM. *p < 0.05, **p < 0.01, ***p < 0.001 by Student’s *t*-test or Mann–Whitney test in accordance with data distribution. One-way ANOVA indicates no significant alteration in WT and KO astrocytes along time. **(E–G)** The graphs depict data from Sholl analysis, reporting the number of intersections of astrocyte processes with concentric circles, in the motor cortex of WT and *Mecp2* KO mice at P20 **(E)**, P40 **(F)**, and P70 **(G)**. Representative images of reconstructed astrocyte arbors by SNT plugin are reported above each graph. *p < 0.05, **p < 0.01, ***p < 0.001 by two-way ANOVA, followed by Sidak’s multiple comparison test. WT and *Mecp2* KO astrocytes (P20: *n* = 36 WT and *n* = 35 KO; P40: *n* = 27 WT and *n* = 44 KO; P70: *n* = 50 WT and *n* = 53 KO; n indicates the number of cells) derived from at least three different animals per genotype (P20: *N* = 4 WT and *N* = 4 KO; P40: *N* = 3 WT and *N* = 5 KO; P70: *N* = 5 WT and *N* = 6 KO; N indicates the number of animals).

**FIGURE 2:**
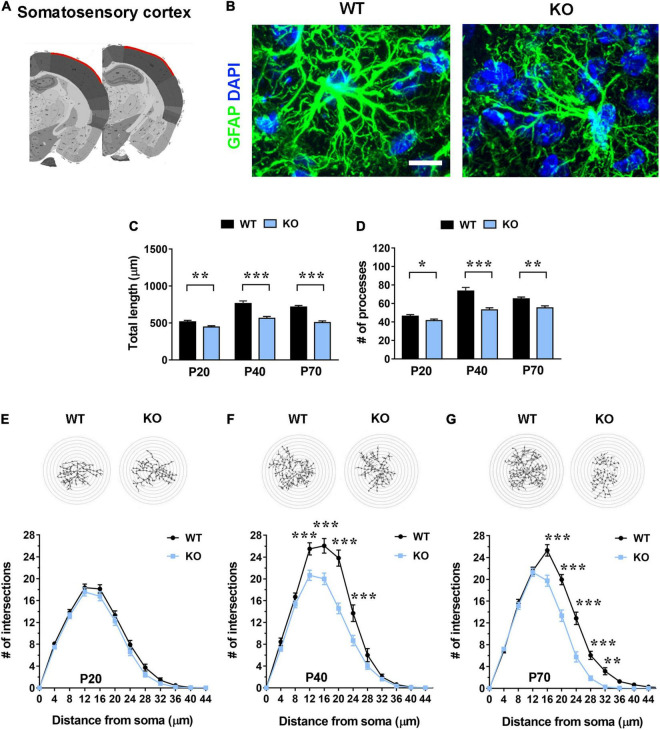
In the somatosensory cortex (layer I) *Mecp2* KO astrocytes show slight cytoskeletal alterations in early-symptomatic mice (P20), that worse in moderate (P40) and severe symptomatic animals (P70). **(A)** Representative images of brain coronal sections, with the somatosensory cortex (layer I) highlighted in red. **(B)** Micrographs are representative images of WT and *Mecp2* KO astrocytes immunostained for GFAP (green) and DAPI (blue) in the somatosensory cortex (layer I) at P40. Scale bar = 10 μm. **(C,D)** The graphs show the total length of processes **(C)** and their number **(D)** in astrocytes in the somatosensory cortex of WT and *Mecp2* KO at P20, P40, and P70. Data are represented as mean ± SEM. *p < 0.05, **p < 0.01, ***p < 0.001 by Student’s *t*-test or Mann–Whitney test in accordance with data distribution. One-way ANOVA indicates a significant increase in the total length and number of WT and KO astrocyte processes from P20 to P40 (*p* < 0.001). **(E–G)** The graphs depict data from Sholl analysis, reporting the number of intersections of WT and KO astrocyte processes with concentric circles, at P20 **(E)**, P40 **(F)**, and P70 **(G)**. Representative images of reconstructed astrocyte arbors by SNT plugin are reported above each graph. **p < 0.01, ***p < 0.001 by two-way ANOVA, followed by Sidak’s multiple comparison test. WT and *Mecp2* KO astrocytes (P20: *n* = 36 WT and *n* = 36 KO; P40: *n* = 16 WT and *n* = 35 KO; P70: *n* = 34 WT and *n* = 36 KO; n indicates the number of cells) derived from at least three different animals per genotype (P20: *N* = 4 WT and *N* = 4 KO; P40: *N* = 3 WT and *N* = 5 KO; P70: *N* = 4 WT and *N* = 4 KO; N indicates the number of animals).

**FIGURE 3:**
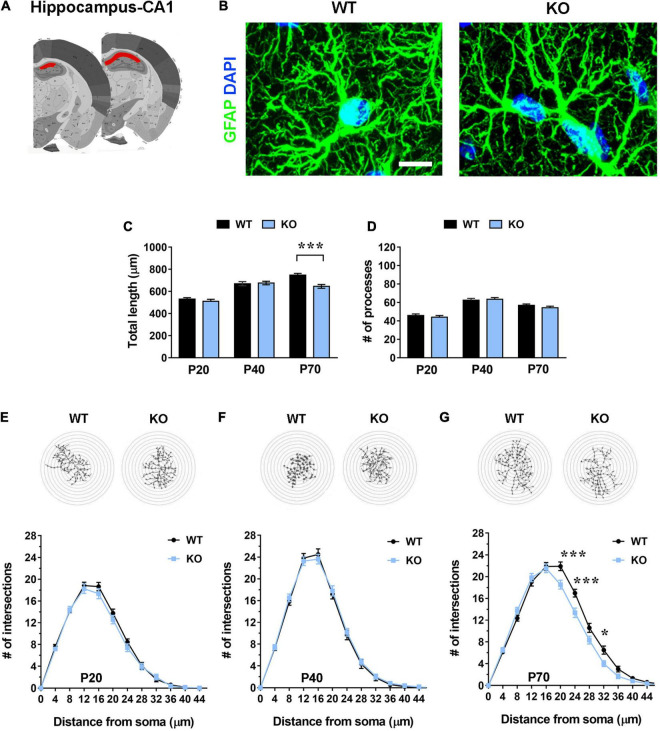
Hippocampal (CA1 pyramidal layer) *Mecp2* KO astrocytes manifest signs of cytoskeletal atrophy only in severe symptomatic animals (P70), but not at earlier time points. **(A)** Representative images of brain coronal sections, with the CA1 of dorsal hippocampus highlighted in red. **(B)** Micrographs are representative images of WT and *Mecp2* KO astrocytes immunostained for GFAP (green) and DAPI (blue) in the CA1 pyramidal layer of the hippocampus at P70. Scale bar = 10 μm. **(C,D)** The graphs show the total length of processes **(C)** and their number **(D)** in *Mecp2* KO astrocytes, compared to WT astrocytes, in the CA1 of dorsal hippocampus of P20, P40, and P70 mice. Data are represented as mean ± SEM. ***p < 0.001 by Student’s *t*-test or Mann–Whitney test in accordance with data distribution. One-way ANOVA indicates a progressive and significant increase in the total length of processes of WT astrocytes (from P20 to P40 *p* < 0.001; from P40 to P70 *p* < 0.01), and an arrest of growth at P40 in KO cells (from P20 to P40 *p* < 0.001). The number of processes increases from P20 to P40 (*p* < 0.001) and slightly decreases at P70 in both WT (*p* = 0.061) and KO (*p* < 0.01) astrocytes. **(E–G)** The graphs depict data from Sholl analysis, reporting the number of intersections of WT and *Mecp2* KO astrocyte processes with concentric circles, at P20 **(E)**, P40 **(F)**, and P70 **(G)**. Representative images of reconstructed astrocyte arbors by SNT plugin are reported above each graph. *p < 0.05, ***p < 0.001 by two-way ANOVA, followed by Sidak’s multiple comparison test. WT and *Mecp2* KO astrocytes (P20: *n* = 36 WT and *n* = 36 KO; P40: *n* = 27 WT and *n* = 45 KO; P70: *n* = 36 WT and *n* = 36 KO; n indicates the number of cells) derived from at least three different animals per genotype (P20: *N* = 4 WT and *N* = 4 KO; P40: *N* = 3 WT and *N* = 5 KO; P70: *N* = 4 WT and *N* = 4 KO; N indicates the number of animals).

**FIGURE 4:**
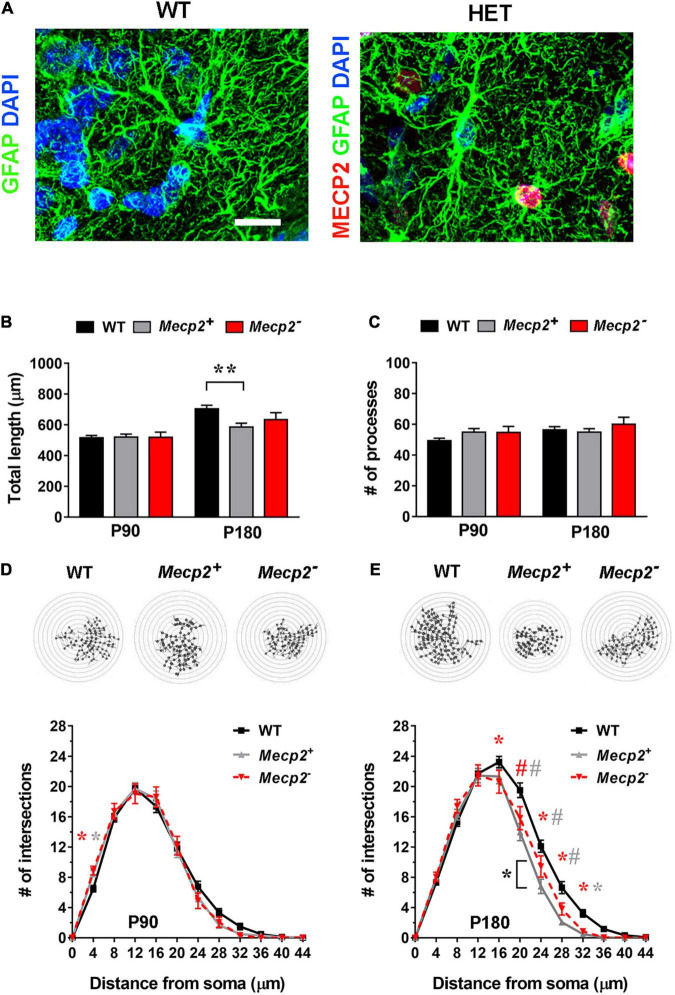
In the layer I of the somatosensory cortex of symptomatic heterozygous animals, the astrocyte cytoskeleton is affected regardless of *Mecp2* expression. **(A)** Micrographs are representative images of astrocytes in the somatosensory cortex (layer I) of WT and heterozygous brains at P180. WT astrocytes were immunostained for GFAP (green) and DAPI (blue); heterozygous brains were also immunostained for Mecp2 (red) to discriminate between cells expressing the WT (*Mecp2*^+^) or null (*Mecp2*^–^) allele. Scale bar = 10 μm. **(B,C)** The graphs show the total length **(B)** and the number of processes **(C)** in Mecp2^+^ (gray) and Mecp2^–^ (red) astrocytes derived from the somatosensory cortex (layer I) of P90 and P180 heterozygous female mice, compared to astrocytes of WT animals (black). Data are represented as mean ± SEM. **p < 0.01 by Kruskal–Wallis test, followed by Dunn’s multiple comparison test. **(D,E)** The graphs depict data from Sholl analysis, reporting the number of intersections of processes of WT (black), Mecp2^+^ (gray) and Mecp2^–^ (red) astrocytes with concentric circles, at P90 **(D)** and P180 **(E)**. Representative images of reconstructed astrocyte arbors by SNT plugin are reported above each graph. *p < 0.05, #*p* < 0.001 by two-way ANOVA, followed by Sidak’s multiple comparison test. * and # indicate the comparison between WT and Mecp2^+^ astrocytes (gray), WT and Mecp2^–^ astrocytes (red), Mecp2^+^ and Mecp2^–^ astrocytes (black). Four animals per genotype were used at each time point (*N* = 4). Astrocytes were randomly selected in the acquired field (P90–110: *n* = 44 WT, *n* = 46 Mecp2^+^, *n* = 18 Mecp2^–^; P180: *n* = 49 WT, *n* = 35 Mecp2^+^ and *n* = 21 Mecp2^–^; n indicates the number of cells).

Mice were anaesthetized by using Tribromoethanol (250 mg/Kg) and transcardially perfused with 4% paraformaldehyde in PBS. Brains were post-fixed 90 min in 4% paraformaldehyde at 4°C and dehydrated in 30% sucrose in PBS at 4°C (∼ 48 h). Brains were frozen in isopentane at −30°C for 3 min and stored at −80°C. Each brain was embedded by PolyFreeze Tissue Freezing Medium (P0091, Merck, Darmstadt, Germany) and sectioning was performed with a cryostat (CM1860, Leica Biosystems, Wetzlar, Germany). Immunofluorescence for GFAP was performed on coronal sections of 40 μm, which were collected in PBS containing 0.1% sodium azide and stored at 4°C. At least three non-consecutive sections for each animal were selected for the analysis in each cerebral region. Representative images are reported in Figure 1A for motor cortex, Figures 2A and 4A for somatosensory cortex, and Figure 3A for hippocampus and at least three animals per group were analyzed. Antigen retrieval was applied before immunostaining, then sections were permeabilized using 0.4% Triton X-100 in PBS for 30 min and blocked with 4% FBS and 0.1% Triton X-100 in PBS for 15 min. Sections were incubated with the primary antibody for GFAP (1:3500; clone GA5, MAB3402, Merck) in blocking solution overnight at 4°C. For immunofluorescence on heterozygous brains, the primary antibody for Mecp2 (1:1000; clone D4F3, #3456, Cell Signaling, Danvers, MA, United States) was also included. Then, sections were washed and incubated with Alexa Fluor anti-mouse 488 and/or anti-rabbit 568 conjugated secondary antibody (1:500; A21202 and A11036, Thermo Fisher Scientific, Waltham, MA, United States) in blocking solution for 1 h in the dark. After several washes in blocking solution (at least five washes of 5 min), DNA was stained with DAPI solution (1:1000 in PBS; #62248, Thermo Fisher Scientific) following a 10-min incubation and sections were washed in PBS. Lastly, they were mounted on microscope slides with Fluoromount Aqueous Mounting Medium (F4680, Merck) and stored at 4°C until image acquisition.

### Microscope Acquisition and Imaging Analysis

For the analysis of GFAP^+^ astrocytes, Z-stack images (212.13 × 212.13 μm^2^, 1,024 × 1,024 pixel resolution, 16-bit grayscale depth) of brain sections were acquired at a Nikon Ti2 Microscope equipped with an A1 + laser scanning confocal system and a Plan Apo λ 60× oil-immersion objective with a step size of 0.5 μm. Digital zoom 1×, offset background, pinhole size, scanning speed, scan direction and line average mode were maintained constant for each dataset acquisition (Supplementary Figures 1, 2). Reconstruction of the arbor of cortical and hippocampal astrocytes was performed using *Simple Neurite Tracer* (SNT), a plugin by Fiji software. Cell selection criteria required for morphometric analyses are described in Tavares et al. (2017). All GFAP^+^ processes of selected astrocytes were manually traced and binarized. Then, the total length of processes and their number were measured with SNT, while *Sholl analysis* plugin was run on the maximum intensity projections to investigate the complexity of astrocytic arbor (Abramoff et al., 2004). Concentric circles spaced by 4 μm and centered on astrocytic nuclei were used.

The cortical thickness of somatosensory and motor cortices was measured in the same sections used for the analysis of GFAP^+^ cells, with additional animals when required, taking the Allen Mouse Brain Atlas as reference. Images from DAPI stained sections were acquired at 4× magnification with a Nikon Eclipse Ni-U epi-fluorescence microscope. Three lines perpendicular to the motor cortex border and five to the somatosensory cortex border were traced along layer I and whole layers and an average of measures was calculated. At least three non-consecutive sections per animal and three animals per group were analyzed and the values obtained were plotted on graph. Only slices between −1.34 mm and −1.82 mm from bregma were included in the analysis. All image analyses were performed by a researcher blind to the genotype.

### RNA Extraction and Quantitative Real Time PCR

Animals (*n* = 8 for each experimental group) were perfused with cold saline solution to remove blood from brain capillaries and brains were isolated and immersed in HBSS solution (H6648, Sigma, Burlington, MA, United States) containing 0.6% glucose. Using a vibratome (VT1000S, Leica Biosystems), 4/5 coronal slices (500 μm) starting from the frontal lobe were obtained from each brain. Slices were maintained in cold 0.6% glucose in HBSS. Under a stereomicroscope motor cortex, somatosensory cortex and dorsal hippocampus were dissected from brain sections accordingly to the Allen Mouse Brain Atlas and immediately frozen on dry ice. In a pilot experiment (data not shown), in order to assess the accuracy in cortical dissection, we analyzed the mRNA levels for genes, which are selectively enriched in the motor (*Ube4b* and *Tnnc1*) and in the somatosensory cortex (*Rorb* and *Tmem215*) (Allen Mouse Brain Atlas).

Total RNA was extracted from all the cerebral regions using PureZOL (#7326890, Bio-Rad, Hercules, CA, United States) and quantified using a NanoDrop spectrophotometer. After assessing RNA integrity by agarose electrophoresis, RNA was reversely transcribed using the RT^2^ First Strand Kit (#330404, Qiagen, Hilden, Germany) as instructed by the manufacturer. The resulting cDNA was used as a template for qRT-PCR with SYBR Green Master Mix (#4472908, Applied Biosystems, Waltham, MA, United States) with designated primers (Table 2). Melting curve showed a single product peak, indicating good product specificity. The best housekeeping gene was selected for each comparison between *CypA and Rpl13*, and fold change in gene expression was calculated using the 2(-delta Ct) method.

**TABLE 2:** The genes and the primers used for qRT-PCR are reported.

Aldh1l1 Aldehyde dehydrogenase 1 family, member L1	Fw: 5′-CAGGAGGTTTACTGCCAGC-3′ Rev: 5′-CACGTTGAGTTCTGCACCCA-3′
AldoC Aldolase, fructose- bisphosphate C	Fw: 5′-TGCTGGGGCTGCTACTGA-3′ Rev: 5′-TCCGCCATCTCCACTGCCT-3′
Aqp4 Aquaporin 4	Fw: 5′-CGGCATCCTCTACCTGGTCACA-3′ Rev: 5′-CAGCGGTGAGGTTTCCAT-3′
CypA Cyclophilin A	Fw: 5′-GGCAAATGCTGGACCAAACACAA-3′ Rev: 5′-GTAAAATGCCCGCAAGTCAAAAG-3′
Chrdl1 Chordin-Like 1	Fw: 5′-TGCCCCAAACTGACCTGTG-3′ Rev: 5′-CATCCGCATGTTCCCACGA-3′
Gfap Glial fibrillary acidic protein	Fw: 5′-ACCCTGGCTCGTGTGGAT-3′ Rev: 5′-ACGTGGACCTGCTGTTGG-3′
Gja1 Gap junction protein alpha 1	Fw: 5′-AGTTCCACCACTTTGGCGTGCC-3′ Rev: 5′-GAGCACCGACAGCCACACCTTC-3′
Gjb6 Gap junction protein beta 6	Fw: 5′-CCTGATCCAACCTACGCTC-3′ Rev: 5′-CACTGCTGCTCTGTTTAGGG-3′
Grm3 Glutamate metabotropic receptor 3	Fw: 5′-GCAGGAGTCATTGGCGGTTCGT-3′ Rev: 5′-GAGTTTGGCACTGGTGGAGGCG-3′
Hapln1 Hyaluronan and proteoglycan link protein 1	Fw: 5′-TGACCACCACCTTTCAGACA-3′ Rev: 5′-GCTTCCACAAGTAGACGGGGGC-3′
Lcat Lecithin-cholesterol acyltransferase	Fw: 5′-AGATCCGTGTCCCTGGCT-3′ Rev: 5′-CGCACTGTCTCATCCCGCA-3′
Mfge8 Milk fat globule EGF and factor 8 protein	Fw: 5′-TGGAACCTGCGTGCTTTTGGCT-3′ Rev: 5′-ACTTGCCTCTGAGTGCCCAGGT-3′
Mertk MER proto-oncogene, tyrosine kinase	Fw: 5′-GCAGTCTTCAGCTGTGAGGCCC-3′ Rev: 5′-GTGGGCGGGGAGGGGATTACTT-3′
Prodh Proline dehydrogenase 1	Fw: 5′-CCCTGCTGTCACGGTTCACT-3′ Rev: 5′-CGGCTGATGGCTGGTTGGAA-3′
Ptprz1 Protein tyrosine phosphatase receptor type Z1	Fw: 5′-CACTGGCGGGAAATGACC-3′ Rev: 5′-ACCATTAGAGCATACGGC-3′
Rpl13 Ribosomal protein L13	Fw: 5′-TGGCTGGCATCCACAAGAAA-3′ Rev: 5′-TTCTTCAGCAGAACTGTCTCCC-3′
Slc1a2 Solute carrier family 1 member 2	Fw: 5′-AGCCAAAGCACCGAAACC-3′ Rev: 5′-TCAGGGTGGATGGGCGAT-3′
Tnc Tenascin C	Fw: 5′-AAAAACAACACCCGAGGC-3′ Rev: 5′-AGGGCTTGAACCAGGTGA-3′

### Statistical Analysis

All data are expressed as mean ± SEM. Before any statistical analysis, normality distribution was evaluated for each dataset by D’Agostino and Pearson test and outliers were assessed by ROUT test (*Q* = 1%) or Grubb’s test (α = 0.05%). Unpaired Student’s *t*-tests or Mann–Whitney tests were used for two group comparisons in accordance with data distribution. To assess the progressive increase in processes number and length of astrocytes and to analyze cortical thickness progression, one-way ANOVA followed by Tukey’s *post hoc* test was applied. A Kruskal–Wallis test followed by Dunn’s *post hoc* test was used to compare astrocytic processes in female brains. Statistical significance for multiple group comparisons was determined by two-way ANOVA, followed by Sidak’s *post hoc* test. All statistical analyses were performed using Prism 8 (GraphPad Software, La Jolla, CA, United States).

## Results

### *Mecp2* KO Astrocytes Show a Reduction in Arbor Complexity, Which Depends on Brain Region and Stage of the Disease

To assess whether the lack of *Mecp2* differently affects astrocyte cytoskeleton with respect to the specific brain region, brain sections from KO animals and WT littermates were stained with an anti-GFAP antibody and shape complexity, total length and number of processes were measured on reconstructed astrocytes (Tavares et al., 2017). Although other methods to resolve the complexity of astrocyte morphology exist, it is recognized that GFAP staining can faithfully be used to reveal the number of primary branches (Figures 1A, 2A, 3A and 4A) (Moye et al., 2019). The analysis was performed on brain areas, whose functional alterations are well documented in RTT, i.e., motor and somatosensory cortices and the CA1 pyramidal layer of dorsal hippocampus (Fukuda et al., 2005; Belichenko et al., 2009; Chapleau et al., 2009; Tropea et al., 2009; Jentarra et al., 2010). Further, our results on pre-synaptic puncta volume demonstrated the presence of smaller spines in the selected cerebral regions of CD1 *Mecp2* null animals, confirming previous data on BL6 mice (Belichenko et al., 2009; Supplementary Figure 3). In the cortical areas the analysis focused on layer I, in line with previous evidence indicating that superficial cortical layers are more affected than deep layers in mutant mice (Fukuda et al., 2005); in addition, GFAP^+^ astrocytes within layer I are scattered and show no overlapping domain, thus facilitating cytoskeletal analysis. Moreover, to the best of our knowledge, a characterization of layer I astrocytes was never reported in RTT and poorly provided in neurodevelopmental disorders in general, although it is recognized that these cortical astrocytes show peculiar gene expression and calcium dynamics (Takata and Hirase, 2008; Bayraktar et al., 2020). To describe cytoskeletal morphology of astrocytes along the disease progression, the analysis was performed at P20, corresponding to an early-symptomatic phase, P40 and P70, representing a moderate and late symptomatic phase, respectively. Analysis of KO astrocytes within the motor cortex revealed the presence of defects already at P20, when RTT behavioral symptoms are not overt, and the maintenance of these defects along the disease progression. Indeed, astroglial cells exhibited a reduction in total length and number of processes at all time points (Figures 1C,D) and accordingly Sholl analysis reported a significant decrease in shape complexity (Figures 1E–G). The same analysis was performed on *Mecp2* null astrocytes of the somatosensory cortex, showing similar defects to those described in the adjacent cortical area. Specifically, at P20, we reported a significant decrease in total length and number of processes of KO astrocytes (Figures 2C,D), although their shape complexity was quite comparable to the WT (Figure 2E). Interestingly, atrophy in KO cells progressed over time, leading to a significant impairment in arbor complexity both at P40 and at P70 (Figures 2F,G). Accordingly, at both ages, we found a reduction in total length of processes, together with defects in their number (Figures 2C,D). Similar alterations were also evident in layer II/III of the cortex of P40 null animals (Supplementary Figure 4), suggesting a widespread impact of *Mecp2* absence on morphological features of GFAP^+^ astrocytes along different cortical layers. On the contrary, in the CA1 pyramidal layer of the hippocampus, cytoskeletal defects were present only at the latest time point (P70), when process ramifications were significantly decreased at 20, 24, and 32 μm from the soma (Figures 3E–G), together with a reduction in total length of branches (Figure 3C), with no effect on their number (Figure 3D).

Further, it is relevant to note the progressive increase of total length and number of astrocyte processes from P20 to P40 in the WT somatosensory cortex, in good accordance with previous data on physiological astrocyte maturation (Holt et al., 2019). Similarly, hippocampal WT astrocytes manifested a significant increase of total length of processes from P20 to P70, whereas KO cells arrested their growth at P40 (Figure 3C).

Interestingly, by analyzing the immunostaining for Mecp2 specifically in GFAP^+^ astrocytes, we observed that its protein levels are higher in layer I of both cortices compared with CA1 hippocampus and that in the somatosensory cortex they overcome those of the motor cortex. Conversely, in neurons Mecp2 is expressed at comparable levels among cortical and hippocampal regions and, accordingly to previous evidence (Ballas et al., 2009; Olson et al., 2014), its expression is almost five-fold higher than that of astrocytes (Supplementary Figure 5).

All in all, our data demonstrate that *Mecp2* KO astrocytes exhibit *in vivo* atrophic signs, that depend on the cerebral region and the age analyzed; further, they suggest that Mecp2 abundance might at least in part contribute to the observed heterogeneity.

### In the Heterozygous Brain Cortical Astrocytes Manifest Atrophic Features Regardless of *Mecp2* Expression

To understand whether non-cell-autonomous mechanisms participate to the observed astrocytic defects, we proceeded analyzing the complexity of GFAP-stained astrocytes in the *Mecp2* heterozygous brains. In particular, we compared astrocytes expressing either the WT or *Mecp2* null allele. Considering the entity of defects in symptomatic null animals, the analysis was performed on layer I of the somatosensory cortex including in the study animals at P90 and P180. Since symptoms in heterozygous animals develop later (Chen et al., 2001; Guy et al., 2001; Cobolli Gigli et al., 2016), we selected these ages as those corresponding to early and moderate symptomatic phases, respectively. WT astrocytes derived from healthy animals of the same litter were used as control.

Analysis at early time point (P90) indicated no alteration in GFAP^+^ processes (Figures 4B–D). Interestingly, at P180, both Mecp2*^+^* and Mecp2*^–^* astrocytes showed a reduced complexity compared to WT (Figure 4E). Intriguingly, Sholl analysis and the measurement of the total length revealed that in the heterozygous brain, astrocytes expressing the WT allele are characterized by a slightly worse phenotype than cells devoid of Mecp2 (Figures 4B,E). Thus, besides confirming the occurrence of astrocyte defects in the heterozygous brain, these data point to the involvement of non-cell autonomous mechanisms in the occurrence of astrocyte cytoskeletal alterations.

### Defects in the Main Processes of *Mecp2* KO Astrocytes Are Accompanied by Reduced Cortical Thickness

**FIGURE 5:**
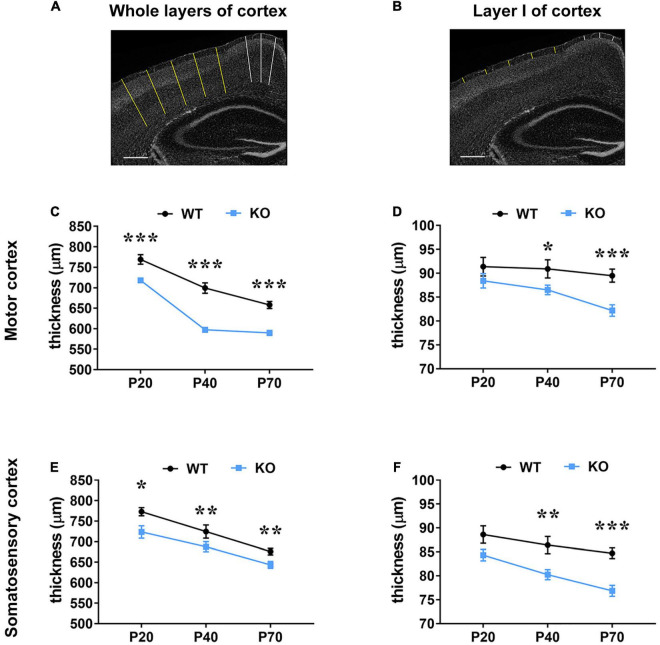
*Mecp2* deficiency causes a reduction of the thickness of the motor and somatosensory cortex, which correlates with astrocytic atrophy. (A,B) Representative images of brain coronal sections stained with DAPI. Traced lines were used to measure the thickness of whole layers (A) and layer I (B) in the motor (white) and somatosensory cortex (yellow). Original magnification: 4×. Scale bar = 400 μm. (C–F) The graphs depict changes in the thickness of whole cortical layers and layer I of the motor (C,D) and somatosensory cortices (E,F) of *Mecp2* null mice compared to their WT littermates, at different ages. Data are represented as mean ± SEM. *p < 0.05, **p < 0.01, ***p < 0.001 by Student’s *t*-test or Mann–Whitney test in accordance with data distribution. One-way ANOVA indicates a significant reduction in the thickness of whole layers of WT and KO motor and somatosensory cortices from P20 to P70 (*p* < 0.001); analysis of layer I reports a significant reduction from P20 to P70 in KO motor (*p* < 0.01) and somatosensory (*p* < 0.001) cortices, but not in WT tissues. Measurements of WT and KO brains derived from at least three non-consecutive slices of at least three different animals per genotype (P20: *N* = 4/5 WT and *N* = 4/6 KO; P40: *N* = 3 WT and *N* = 5 KO; P70: *N* = 5 WT and *N* = 5 KO).

Microcephaly is one of the RTT hallmarks and a progressive reduction of the thickness of the motor and somatosensory cortices has been reported in the *Mecp2* null model in C57BL/6 background, compared to age-matched animals (Fukuda et al., 2005). Thus, we proceeded by assessing whether a similar defect is present in the same mutant animals maintained in the outbred CD1 background, which have been used throughout this study, and whether a possible correlation exists between astrocytic cytoskeletal features and cortical defects. Our data confirmed the presence of a reduced thickness of the motor and somatosensory cortices also in *Mecp2* deficient CD1 mouse model, compared to the corresponding age-matched WT from the same litter (Figure 5). In particular, in KO animals we observed a significant reduction of the thickness of whole layers of the motor and somatosensory cortices at all ages analyzed (Figures 5A,C,E). Conversely, the analysis of the thickness of layer I reported a significant decrement only at P40 and P70 (Figures 5B,D,F).

### Transcriptional Alterations of Astrocyte-Enriched Genes in *Mecp2* KO Brains Confirm a Regional and Temporal Astrocyte Heterogeneity

The described astrocyte atrophy might contribute to functional alterations, which could originate from and/or lead to transcriptional defects (Verkhratsky et al., 2014; Schiweck et al., 2018). We thus used qRT-PCR to assess whether *Mecp2* KO astrocytes suffer from transcriptional defects, which could differ depending on the cerebral regions and the disease progression. Instructed by our previous analyses, we selected for our studies a time point characterized by the absence or presence of mild morphological defects in KO astrocytes (i.e., P20 for motor and somatosensory cortex and P40 for the hippocampus), and a symptomatic time point, corresponding to P40 for the cortical samples and P70 for the hippocampus. To circumvent the limit of using samples containing multiple cell populations, we analyzed the expression of 16 genes selected from two comprehensive lists of transcripts predominantly expressed by astroglial cells (Cahoy et al., 2008; Zhang et al., 2014), also considering their function and putative involvement in RTT or other intellectual disabilities. We grouped them according to their biological functions: astrocytic markers (*Gfap, Aldoc*, and *Aldh1l1*), intercellular communication (*Gjb6* and *Gja1*), homeostasis (*Aqp4, Scl1a2*, and *Grm3*), synapse formation and elimination (*Chrdl1, Mfge8*, and *Mertk*), extracellular matrix components (*Ptprz1, Hapln1*, and *Tnc*), lipid metabolism (*Lcat*) and mitochondrial metabolism (*Prodh*).

**FIGURE 6:**
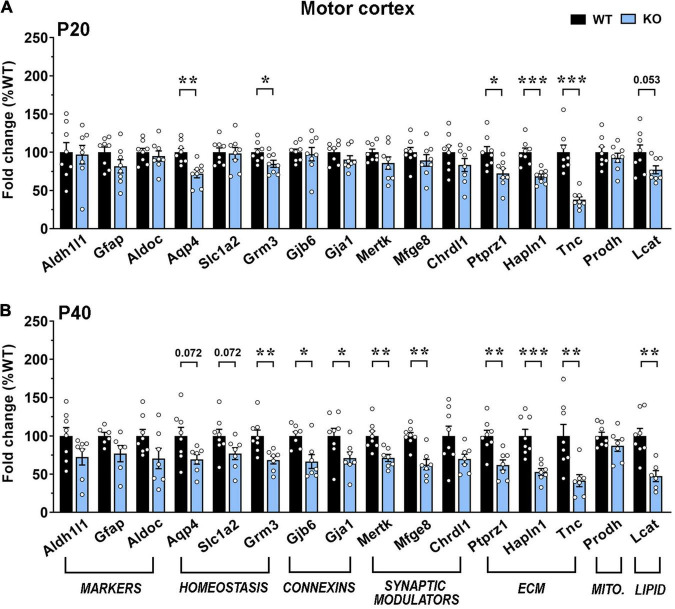
Transcriptional expression of astrocytic genes in the motor cortex of early-symptomatic and moderate symptomatic *Mecp2* KO animals. (A,B) The graphs depict the mRNA expression of selected astrocytic genes in motor cortex of early-symptomatic [at P20 (A)] and moderate symptomatic [at P40 (B)] KO mice, compared to the corresponding WT littermates. Data are represented as mean ± SEM. *p < 0.05; **p < 0.01; ***p < 0.001 by Student’s *t*-test or Mann–Whitney test in accordance with data distribution. Eight animals per genotype were used at each time point (*N* = 8).

**FIGURE 7:**
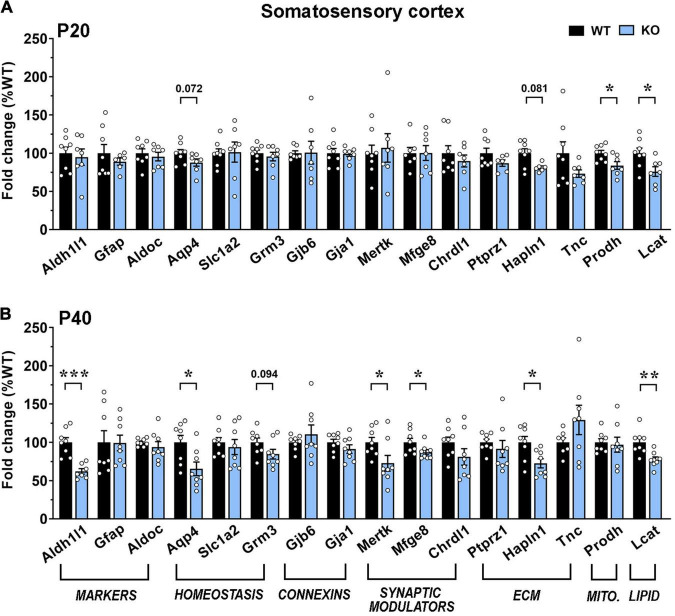
Transcriptional expression of astrocytic genes in the somatosensory cortex of early-symptomatic and moderate symptomatic *Mecp2* KO animals. (A,B) The graphs depict the mRNA expression of selected astrocytic genes in somatosensory cortex of early-symptomatic [at P20 (A)] and moderate symptomatic [at P40 (B)] KO mice, compared to the corresponding WT littermates. Data are represented as mean ± SEM. *p < 0.05; **p < 0.01; ***p < 0.001 by Student’s *t*-test or Mann–Whitney test in accordance with data distribution. Eight animals per genotype were used at each time point (*N* = 8).

Our results indicate an overall transcriptional decrement, which depends on the brain region and the disease progression. In details, in the motor cortex, we reported a significant downregulation of 5 genes (*Aqp4, Grm3, Ptprz1, Hapln1*, and *Tnc*) and a trend toward a decrement for *Lcat* in KO at P20. At P40, transcription of most genes was reduced, with 9 of them exhibiting a significant decrement. Interestingly, at both ages, mRNA levels of *Grm3*, *Lcat* and genes encoding for extracellular matrix components were reduced, whereas astrocytic marker genes, *Chrdl1* and *Prodh* were not affected. Conversely, the P40 KO motor cortex exhibited a significant defect in the expression of genes involved in astrocyte communication (connexins) and synapse remodeling (Figures 6A,B). A similar progressive transcriptional defect was observed in the KO somatosensory cortex, that however, involved different genes. Indeed, only 4 genes (*Aqp4, Hapln1, Prodh*, and *Lcat*) were found deregulated at P20, whilst almost half of the selected ones (*Aqp4, Hapln1, Lcat*, *Aldh1l1, Grm3, Mertk*, and *Mfge8*) were affected at P40. Only *Prodh* recovered its expression at P40 (Figures 7A,B).

**FIGURE 8:**
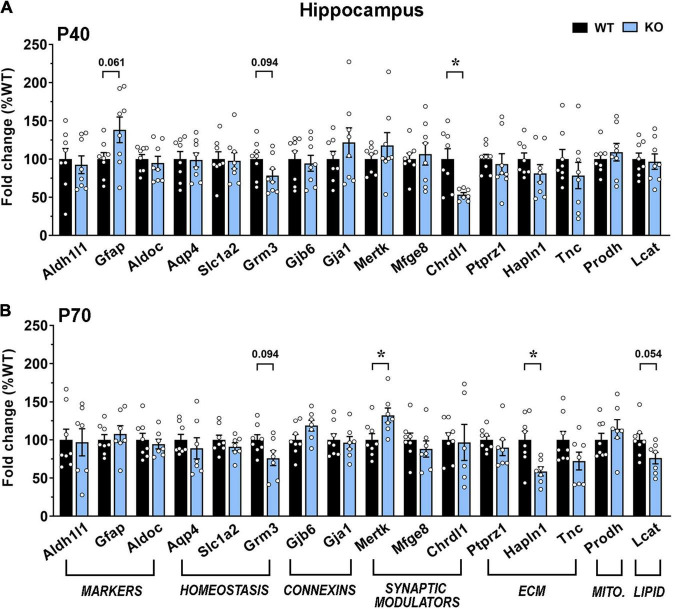
Transcriptional expression of astrocytic genes in the dorsal hippocampus of moderate and severe symptomatic *Mecp2* KO animals. (A,B) The graphs depict the mRNA expression of selected astrocytic genes in motor cortex of moderate [at P40 (A)] and severe symptomatic [at P70 (B)] KO mice, compared to the corresponding WT littermates. Data are represented as mean ± SEM. *p < 0.05 by Student’s *t*-test or Mann–Whitney test in accordance with data distribution. Eight animals per genotype were used at each time point (*N* = 8).

In good accordance with the morphological analyses, transcription in KO hippocampus was less affected. Indeed, at the early time point, only *Chrdl1* was significantly downregulated, while at P70, *Mertk* and *Hapln1* were significantly downregulated and *Lcat* showed a trend toward a reduction (Figures 8A,B).

## Discussion

Traditionally, a neurocentric view focusing on neurons and their connectivity has been widely applied to RTT, with an emphasis on the effects of *MECP2* mutations on neuronal maturation, synaptogenesis and functioning. However, over the past 10 years, it gradually emerged the involvement of astrocytes in RTT as well as in other neurodevelopmental disorders, such as Fragile X and Down syndrome (Cresto et al., 2019). Recent findings have also associated MeCP2 with neurodegenerative diseases, including Alzheimer’s, Parkinson’s, and Huntington’s diseases (Ausió et al., 2014), in which astrocytes undergo atrophy and asthenia affecting their homeostatic and protective functions (Verkhratsky and Nedergaard, 2018).

Herein, we demonstrate the presence of atrophic features in *Mecp2* deficient astrocytes in the motor and somatosensory cortices (layer I) and CA1 area of dorsal hippocampus, indicating the occurrence of a progressive reduction in the complexity of mutant astrocytes, with a different temporal profile depending on the brain area, highlighting a regional heterogeneity. Additionally, in the heterozygous brain, astrocytes show a cytoskeletal impairment regardless of which *Mecp2* allele is expressed, with more pronounced defects in Mecp2^+^ cells. Finally, transcriptional analysis of selected astrocyte-enriched genes proves a general downregulation, which deteriorates with time, suggesting a global defect in astrocyte maturation and functionality.

In this study, we focused our analyses on cerebral regions known to be involved in RTT pathology (Fukuda et al., 2005; Belichenko et al., 2009; Chapleau et al., 2009; Tropea et al., 2009; Jentarra et al., 2010). Regarding the analysis of cytoskeleton of cortical astrocytes, we selected GFAP^+^ cells in layer I, which plays a crucial role in development, maturation and function of the cortex (Trutzer et al., 2019) and which contains many astrocytes with high level of GFAP, smaller volume and different features compared to other layers (Lanjakornsiripan et al., 2018). We reported that, while in the motor cortex KO astrocytes display cytoskeletal alterations already during the early symptomatic stage with persistence of defects along time, in the somatosensory cortex astrocytes manifest a progressive impairment from P20 to P40, that was maintained at P70. Conversely, in the hippocampus, *Mecp2* deficiency induces an arrest of cytoskeletal growth only at later time points. Our results are in good accordance with previous data, demonstrating the presence of atrophic features with fewer and poorly branched ramifications of astrocytes in corpus callosum and dentate gyrus of the *Mecp2*^308/y^ mouse model (De Filippis et al., 2012). Similarly, the conditional deletion of *Mecp2* in late juvenile animals induced hippocampal astrocytes to show a minor process complexity 18 weeks after deletion (Nguyen et al., 2012). In accordance with our results, analysis on human brains indicated a less intense and widespread immunoreactivity of GFAP in RTT samples (Pejhan et al., 2020) and human *MECP2* mutant iPSC-derived astrocytes display microtubule defects, indicative for cytoskeletal abnormalities (Delépine et al., 2016).

The molecular mechanisms behind cytoskeletal impairment in *Mecp2* deficient astrocytes are not understood, but some data point to defects in microtubules, which coextend with intermediate filaments to form the main processes (De Filippis et al., 2012; Nectoux et al., 2012). Indeed, an increased microtubule growth rate, indicative of a more destabilized state of cytoskeleton, was reported in astrocytes cultured from either *Mecp2*^308/y^ or *Mecp2* null animals, and a pharmacological intervention targeting Rho-GTPase, a group of enzymes implicated in cytoskeleton dynamics, reversed astrocyte atrophy (De Filippis et al., 2012; Nectoux et al., 2012). Additionally, HDAC6 inhibition rescued microtubule dynamics in *Mecp2* null astrocytes, concomitantly with an amelioration of behavioral outcomes in *Mecp2* mutant animals (Lebrun et al., 2021). Further, molecular data highlighted the deregulation of a set of genes/proteins associated with cytoskeletal structure, maturation and morphology in the cortex of symptomatic *Mecp2* KO mice (Pacheco et al., 2017).

Although several neurobiological defects have been reported in RTT animal models and patients, a description of their progression along brain development has been provided only for few of them. For instance, a characteristic feature of RTT brain is microcephaly and Fukuda et al. (2005) measured the cortical thickness, neuronal size and density as well as synaptic features in the somatosensory cortex of *Mecp2* KO animals at different ages, demonstrating a delay in cortical maturation. Besides confirming in *Mecp2* KO CD1 mouse the reduction of the thickness of whole cortical layers, we added novel data about the thickness of layer I, demonstrating a worsening along time; further, we noticed in WT animals a progressive reduction of whole cortical thickness along with brain maturation, in accordance with MRI data (Hammelrath et al., 2016). Interestingly, we observed a temporal correlation between the progressive thinning of layer I and the atrophy of astrocytes. Thus, although reduced cortical thickness has been mainly attributed to immature neurons with less and smaller spines (Fukuda et al., 2005; Belichenko et al., 2009; Supplementary Figure 3), we suggest that cytoskeletal atrophy in astrocytes might contribute to layer I cortical defects.

Importantly, morphological alterations in astrocytes can negatively impact their proper functions, thus leading, as a consequence, to neuronal dysfunctions and synaptic defects. Indeed, *in vitro* experiments reported aberrant calcium signaling in *Mecp2* null astrocytes and their inability to correctly support neuronal maturation (Ballas et al., 2009; Dong et al., 2018). Similar to RTT, strong evidence exists for neurodegenerative disorders in which astrocyte alterations impinge on neuronal health, affecting both morphological and functional features (Finsterwald et al., 2015). In order to have an insight on the functionality of *Mecp2* deficient astrocytes, we analyzed the transcriptional expression of astrocyte-enriched genes associated with specific functions. As previously observed in RTT models (Li et al., 2013; Bedogni et al., 2014), our data point toward a global downregulation of transcription, which depends on the cerebral region and the disease stage. Considering that the expression of astrocytic markers is preserved in KO brains, the observed decrement in gene expression might not be attributed to a reduced number of astrocytes but rather to a defective maturation, in accordance with previous data (Pacheco et al., 2017) and as already reported for null neurons (Cobolli Gigli et al., 2018). Whatever the reason, the broad-spectrum of molecular alterations is indicative of a general impairment in astrocyte functionality.

Several studies reported heterogeneity of astrocytes in terms of form and functions, both between and within cerebral regions (Haim and Rowitch, 2017; Ohlig et al., 2021). Thus, we considered important to characterize astrocyte cytoskeletal structure and molecular features in more than one cerebral area, reporting a heterogeneity of phenotypes among different brain regions.

The different onset and entity of astrocytic defects in *Mecp2* mutant brains in relation to the region residency might arise from different mechanisms, considering that astrocyte heterogeneity relies both on intrinsic signals and neuronal cues (Haim and Rowitch, 2017; Lanjakornsiripan et al., 2018). Astrocytic defects might directly depend, at least in part, on *Mecp2* deficiency. In that case, phenotype might correlate with levels of the methyl binding protein. In possible good accordance, our data report that WT astrocytes express higher Mecp2 protein levels in layer I of both somatosensory and motor cortex with respect to hippocampus. However, considering that the Mecp2 deficient brain suffers from complex molecular and functional defects, we believe that other indirect factors can participate to the described astrocyte heterogeneity. For instance, we can suggest that a diverse signature of DNA methylation might play a role in the heterogeneity among inter-regional astrocytes, as reported for cortical and cerebellar astrocytes (Welle et al., 2021). Further, considering that a plethora of different post-translational modifications (PTMs) generates and regulates the functional versatility of MeCP2 (Bellini et al., 2014), future studies should also investigate if different events of PTM affect Mecp2 functions in astrocytes. In parallel, alterations in neuronal activity, commonly detected in Mecp2 mutant brains, could reasonably affect astrocyte morphology and maturation.

Eventually, it is well recognized that biologically significant phenotypes observed in *Mecp2* null mice should be confirmed in female heterozygous models, which best represent the human condition (Katz et al., 2012). We thus included in our study the analysis in the heterozygous cortex, where we found that astrocytes display signs of cytoskeletal atrophy independently from *Mecp2* expression, suggesting the occurrence of non-cell autonomous effects from *Mecp2*^–^ to *Mecp2*^+^ cells. Actually, the existence of non-cell autonomous mechanisms among RTT astrocytes has been already demonstrated and gap junctions-mediated mechanisms have been implicated (Maezawa et al., 2009). However, since we used a heterozygous animal, we cannot rule out that non-cell autonomous effects could be mediated by other cells beyond astrocytes, a possibility that could be excluded analyzing astrocytes in a conditional heterozygous animal lacking *Mecp2* exclusively in these glial cells.

To conclude, our data describe the occurrence and progression of cytoskeletal and molecular alterations in astrocytes from *Mecp2* mutant animals, underlying once again the involvement of this cell population in the pathogenesis of RTT. Considering their role in brain communication and higher brain functions, such as cognition, and the fact that conditional re-expression of *Mecp2* in astrocytes improves behavioral outcomes (Lioy et al., 2011), astrocytes represent an interesting therapeutic target in RTT. However, our results suggest that the development of efficacious therapeutic strategies should consider astrocytic heterogeneity and the diversity of phenotypes, which affect these glial cells along the progression of the disease.

## Data Availability Statement

The raw data supporting the conclusions of this article will be made available by the authors, without undue reservation.

## Ethics Statement

The animal study was reviewed and approved by the OPBA (Animal Welfare Organism) of University of Milan.

## Author Contributions

AF, NL, and EA contributed to the conception and design of the study, and interpreted the results. EA performed most of the experiments and all statistical analysis of data. EF and FM contributed to imaging analysis. IS contributed to molecular study. AF supervised all the experiments and analysis of data. AF and EA wrote the manuscript. NL revised the manuscript. All authors contributed to manuscript revision, read, and approved the manuscript.

## Conflict of Interest

The authors declare that the research was conducted in the absence of any commercial or financial relationships that could be construed as a potential conflict of interest. The handling editor declared a shared affiliation, though no other collaboration with several of the authors IS and NL at the time of the review.

## Publisher’s Note

All claims expressed in this article are solely those of the authors and do not necessarily represent those of their affiliated organizations, or those of the publisher, the editors and the reviewers. Any product that may be evaluated in this article, or claim that may be made by its manufacturer, is not guaranteed or endorsed by the publisher.
