# Supplementary material for: Identification of Region-Specific Cytoskeletal and Molecular Alterations in Astrocytes of Mecp2 Deficient Animals
Source: Front Neurosci. 2022 Feb 15;16:823060. doi: 10.3389/fnins.2022.823060 (PMC8886113; doi:10.3389/fnins.2022.823060)
Supplement: Supplementary file 3 [file Image_1.pdf]

Supplementary materials

Supplementary figure 1

Representative z-stack acquisition of a brain section for the analysis of GFAP$^+$ astrocytes. Images were acquired at a laser scanning confocal microscope with a Plan Apo λ 60x oil-immersion objective and a step size of 0.5 µm. In the somatosensory cortex, astrocytes with GFAP-positive processes (green) and their nuclei (blue) are visualized.

Supplementary figure 2

Representative z-stack acquisition of a brain section for the analysis of *Mecp2*$^+$ and *Mecp2*$^-$ GFAP$^+$ astrocytes. Images were acquired at a laser scanning confocal microscope with a Plan Apo λ 60x oil-immersion objective and a step size of 0.5 µm. In the somatosensory cortex, *Mecp2*$^+$ (red) and *Mecp2*$^-$ astrocytes with GFAP-positive processes (green) and their nuclei (blue) are visualized.

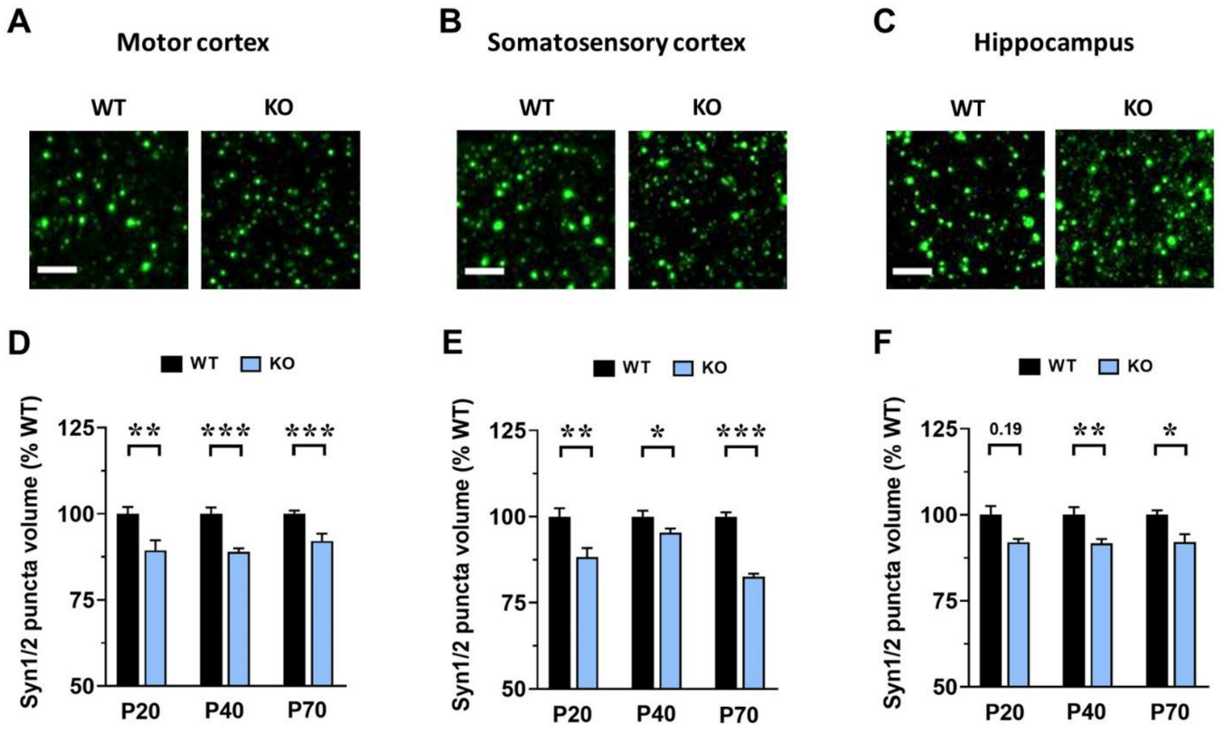

Supplementary figure 3

***Mecp2* KO neurons in the motor and somatosensory cortex (layer I) and in the CA1 pyramidal layer of the hippocampus exhibit pre-synaptic alterations (A,B,C).** Representative immunostaining for Synapsin1/2 (green) of WT and *Mecp2* KO mice at P20 in the layer I of the motor (A) and somatosensory cortex (B), and at P40 in the dorsal hippocampus (C). Scale bar: 3 µm.

(D,E,F) The graphs show the percentages of the mean volume of Synapsin1/2 puncta compared to WT animals (100%). Data are represented as mean ± SEM. *p<0.05, **p<0.01, ***p<0.001 by Student's t-test or Mann-Whitney test in accordance with data distribution. Measurements derived from at least 3 slices of at least 3 different animals per genotype (P20: N=3/5 WT and 3/4 KO; P40: N=3/5 WT and 3/5 KO; P70: N=4 WT and 3 KO).

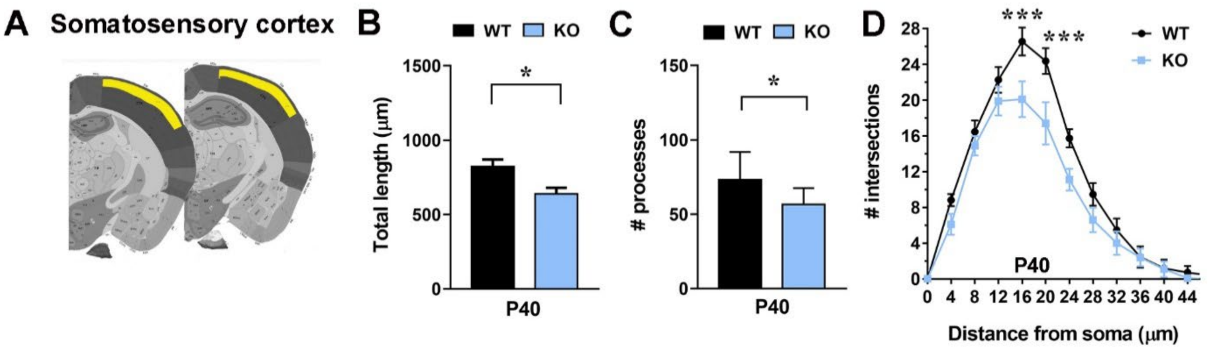

Supplementary figure 4

***Mecp2* KO astrocytes in the layer II/III of the somatosensory cortex show atrophic features at P40 suggesting a widespread impact of Mecp2 absence on GFAP$^+$ astrocytes. (A)** Representative images of brain coronal sections, with the layer II/III of the somatosensory cortex highlighted in yellow. **(B,C)** The graphs show the total length of processes (B) and their number (C) in WT and *Mecp2* KO astrocytes in the layer II/III of the somatosensory cortex at P40. Data are represented as mean ± SEM. *p<0.05 by Student's t-test. **(D)** The graph depicts data from Sholl analysis, reporting the number of intersections of WT and KO astrocyte processes with concentric circles. ***p<0.001 by two-way Anova, followed by Sidak's multiple comparison test. WT and *Mecp2* KO astrocytes (n=11 WT and n=10 KO; n indicates the number of cells) derived from at least 3 different animals per genotype (N=3 WT and N=5 KO; N indicates the number of animals).

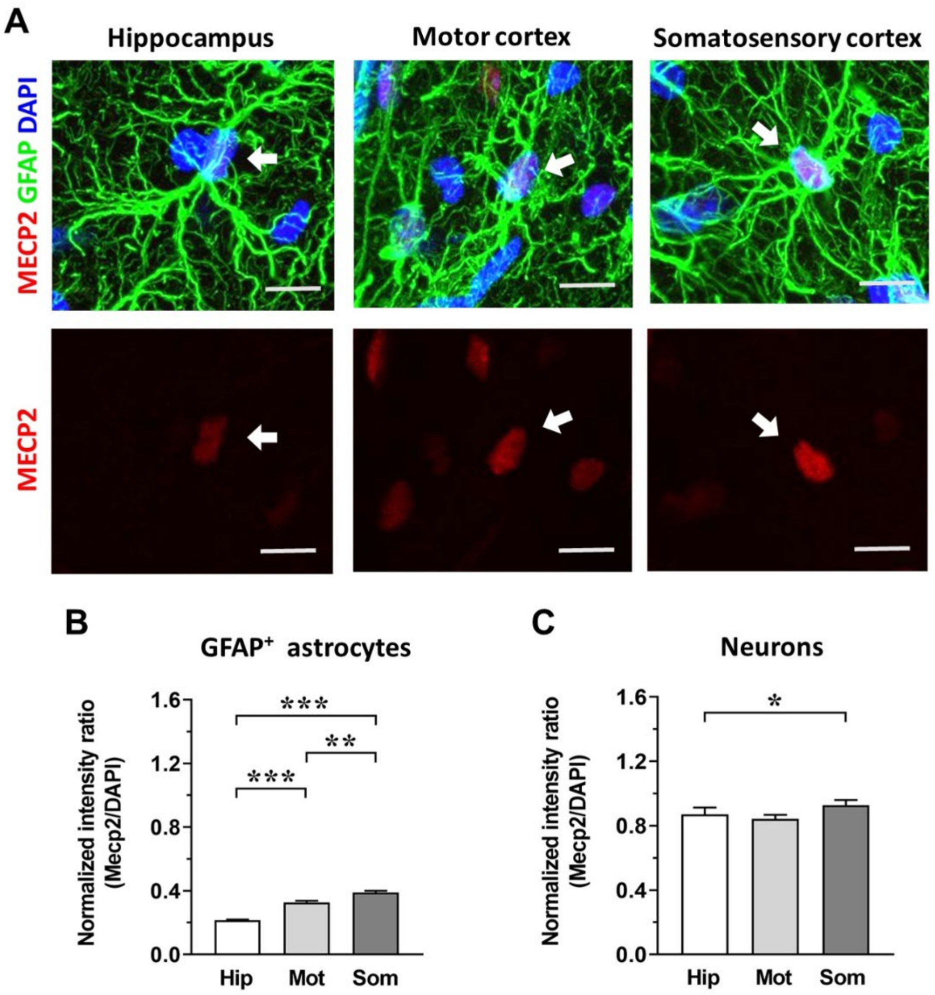

Supplementary figure 5

In somatosensory and motor cortex (layer I) GFAP$^+$ astrocytes express higher Mecp2 levels compared to hippocampal astrocytes. (A) Micrographs are representative images of astrocytes (indicated by arrows) immunostained for GFAP (green), Mecp2 (red) and DAPI (blue) in the layer I of the motor and somatosensory cortex, and in the CA1 area of the dorsal hippocampus in WT animals at P40. Scale bar: 10 µm. (**B,C**) The graphs show the integrated density of Mecp2 fluorescent signal normalized on DAPI staining. Data are represented as mean ± SEM. *p<0.05, **p<0.01, ***p<0.001 by Kruskal-Wallis test, followed by Dunn's multiple comparison test. GFAP$^+$ astrocytes were analysed in layer I of the motor (Mot) and somatosensory cortex (Som), and in the CA1 region of the dorsal hippocampus (Hip) (Mot: n=102; Som: n=118; Hip: n=175; n indicates the number of cells). Neurons were analysed in the layer II/III of the motor and somatosensory cortex, and in the CA1 pyramidal layer of the hippocampus (Mot: n=96; Som: n=94; Hip: n=102; n indicates the number of cells). Both astrocytes and neurons derived from 4 different slices of 3 different WT animals (N=3).

Immunofluorescence for Synapsin1/2

Brains collected for the analysis of astrocyte cytoskeleton and cortical thickness were also used for pre-synaptic immunostaining. Immunofluorescence for Synapsin1/2 was performed on coronal sections of 40 μm, which were collected in PBS containing 0.1% sodium azide and stored at 4°C. At least three non-consecutive sections for each animal were selected for the analysis in each cerebral region (representative images are reported in panel A,B,C of Supplementary figure 4 for motor cortex, somatosensory cortex and dorsal hippocampus, respectively) and at least three animals per group were analyzed. Sections were permeabilized and blocked with 10% horse serum and 0.5% Triton X-100 in PBS for 1 hour. Then, they were incubated with the primary antibody for Synapsin1/2 (1:500; #106006, Synaptic Systems) in incubation solution (3% horse serum and 0.5% Triton X-100 in PBS) overnight at 4°C. Sections were washed and incubated with Alexa Fluor anti-chicken 488 conjugated secondary antibody (1:500; A32931, Thermo Fisher) in incubation solution for 1 hour and 30 minutes in the dark. After several washes in incubation solution (at least 6 washes of 10 minutes), DNA was stained with DAPI solution (1:1000 in PBS; #62248, Thermo Fisher) following a 10-minute incubation and sections were washed in PBS. Lastly, they were mounted on microscope slides with Fluoromount Aqueous Mounting Medium (F4680, Merck) and stored at 4°C until image acquisition.

Microscope acquisition and imaging analysis for pre-synaptic puncta

Z-stacks images (127.28 × 127.28 μm^2, 1,024 × 1,024 pixel resolution, 16-bit grayscale depth) were acquired at a Nikon Ti2 Microscope equipped with an A1+ laser scanning confocal system and a SR Apo TIRF 100x oil-immersion objective with a step size of 0.3 μm. A minimum of 15 stacks/image were acquired. Digital zoom, offset background, pinhole size, scanning speed, scan direction and line average mode were maintained constant for each dataset acquisition. Analysis of puncta volume was performed with Arivis 4D Vision software (Arivis, AG, München, Germany), that allowed a 3D rendering of the immunostaining. Briefly, *Attenuation correction* plugin by Fiji software was run on all Z-stacks images to correct fluorescence intensity decrease along depth. Then, a specific pipeline was created with Arivis to process and segmentate each image, obtaining the reconstructed puncta volume. To analyse different fields of the same brain section and reduce any internal variability (i.e. presence of nuclei or blood vessels), 6 ROIs (30 μm x 30 μm) were designed in non-overlapping positions. To do this, we wrote a python script to create the sub-volumes boxes matrix. The average value of the reconstructed volume of Synapsin1/2 puncta of each ROI was used for statistical analysis.

Immunofluorescence for Mecp2 expression in astrocytes and neurons

WT brains collected at P40 for the analysis of astrocyte cytoskeleton and cortical thickness were also used for Mecp2 immunostaining. Immunofluorescence for GFAP and Mecp2 was performed as

previously reported (see Methods and Materials). To discriminate neuronal cells, the primary antibody for NeuN (1: 1000; clone A60, MAB377, Merck) and Alexa Fluor anti-mouse 488 conjugated secondary antibody (1:500; A21202, Thermo Fisher) were used.

Microscope acquisition and imaging analysis for Mecp2 expression in astrocytes and neurons

Z-stack images of brain sections were acquired following the same protocol used to analyze astrocyte cytoskeleton. All acquisition parameters for Mecp2 and DAPI were maintained constant for each brain area and animal. To perform the analysis of Mecp2 fluorescence intensity in astrocytes and neurons, cells were discriminated by GFAP or NeuN staining, respectively. Cells were randomly selected along all stacks and a ROI was manually traced around their nuclei on DAPI channel. Measurements of Mecp2 integrated density were performed with Fiji software. To overcome the eventual decrease of the fluorescent signal along depth, we decided to normalize Mecp2 value on the intensity of DAPI staining for each cell. The normalized intensity ratio was used for statistical analysis. At least six astrocytes and neurons per slices were analysed and four slices per animals were used.
